# Carbonation and Corrosion Durability Assessment of Reinforced Concrete Beam in Heavy-Haul Railways by Multi-Physics Coupling-Based Analytical Method

**DOI:** 10.3390/ma18153622

**Published:** 2025-08-01

**Authors:** Wu-Tong Yan, Lei Yuan, Yong-Hua Su, Long-Biao Yan, Zi-Wei Song

**Affiliations:** 1 Railway Engineering Research Institute, China Academy of Railway Sciences Corporation Limited, Beijing 100081, China; yanwutong@bjtu.edu.cn (W.-T.Y.); rainycars@126.com (L.Y.); 2 State Key Laboratory of High Speed Railway Track System, China Academy of Railway Sciences Corporation Limited, Beijing 100081, China; 3 Beijing Urban Construction Group Co., Ltd., Beijing 100088, China; yanlongbiao@bjtu.edu.cn; 4 School of Civil Engineering, Beijing Jiaotong University, Beijing 100044, China; zw.song@bjtu.edu.cn

**Keywords:** concrete carbonation, reinforcement corrosion, reinforced concrete beams, durability assessment, multi-physics field coupling

## Abstract

The operation of heavy-haul railway trains with large loads results in significant cracking issues in reinforced concrete beams. Atmospheric carbon dioxide, oxygen, and moisture from the atmosphere penetrate into the beam interior through these cracks, accelerating the carbonation of the concrete and the corrosion of the steel bars. The rust-induced expansion of steel bars further exacerbates the cracking of the beam. The interaction between environmental factors and beam cracks leads to a rapid decline in the durability of the beam. To address this issue, a multi-physics field coupling durability assessment method was proposed, considering concrete beam cracking, concrete carbonation, and steel bar corrosion. The interaction among these three factors is achieved through sequential coupling, using crack width, carbonation passivation time, and steel bar corrosion rate as interaction parameters. Using this method, the deterioration morphology and stiffness degradation laws of 8 m reinforced concrete beams under different load conditions, including those of heavy and light trains in heavy-haul railways, are compared and assessed. The analysis reveals that within a 100-year service cycle, the maximum relative stiffness reduction for beams on the heavy train line is 20.0%, whereas for the light train line, it is only 7.4%. The degree of structural stiffness degradation is closely related to operational load levels, and beam cracking plays a critical role in this difference.

## 1. Introduction

Concrete carbonation and steel reinforcement corrosion are prominent types of damage that occur in reinforced concrete (RC) beams under normal atmospheric conditions [1,2,3]. This damage is accompanied by phenomena such as concrete cracking, steel bar corrosion, as well as swelling and spalling of the protective layer. These common deficiencies result in degradation of the beam’s stiffness, which may lead to significant issues during its service life [4].

To date, extensive research has been conducted on the carbonation mechanism and depth prediction of concrete structures [5,6]. However, most studies focused on the carbonation depth analysis of undamaged concrete [7]. Papadakis et al. [5,6] conducted in-depth research on the carbonation mechanism of concrete and proposed a representative formula for calculating carbonation depth. Based on this mathematical model, Park [8] established a finite element method for simulating the carbonation process of concrete. This work made it applicable to the carbonation depth analysis of concrete structures that were not convenient for analytical solutions. The process of concrete carbonation was complicated and influenced by various factors, including material properties, exposure conditions, and construction details [9,10]. Li [11] pointed out that the carbonation rate of concrete reaches its maximum when the relative humidity is 55%. The equations of Papadakis et al. [5,6] were applicable for cases of relative humidity between 50% and 90% and overestimated the carbonation depth for low-humidity cases below 50%. Li [11] proposed a modified equation for Papadakis et al. [5,6] to consider different humidity conditions. Overall, the carbonation problem of concrete materials has been well studied, and the calculation formula for the carbonation depth is provided in the design code [12]. Recent progress has focused on new construction materials [13], such as carbonation and chloride ion penetration of lightweight aggregate concrete and high-performance concrete.

However, the previously presented studies mainly focused on the carbonation of undamaged concrete. During the service life, cracking in concrete structures is inevitable, which may be caused by factors such as shrinkage, temperature fluctuations, or applied loads. Ghantous et al. [14] demonstrated through experimental studies that concrete at crack locations undergoes accelerated carbonation, leading to preferential corrosion initiation in the adjacent steel reinforcement. In recent years, accelerated carbonation in cracked concrete has gradually emerged as a research hotspot [15,16,17]. The tests by Schutter et al. [18] showed that the mean carbonation depth was 22 mm for uncracked zones, but increased to 34 mm for cracked zones with a crack width of 0.12 mm. The tests by Alahmad et al. [19] showed that for crack widths of 60 μm or wider, the perpendicular-to-crack carbonation depths are similar to the surface carbonation depth. Han et al. [20] assumed the diffusion coefficient of CO_2_ at open cracks to be 1000 times that of the diffusion coefficient within the concrete. These methods did not establish a clear quantitative relationship between crack parameters and the accelerated diffusion coefficient. Creazza et al. [21] proposed a formula for the gas diffusion amplification coefficient based on concrete damage in their study; however, this formula has not been applied to concrete structures in practice. Shi et al. [22,23,24] followed the formula form by Creazza et al. [21] but modified the coefficient values. Then, this formula was employed to analyze the carbonation behavior of a cracked concrete specimen using a meso-scale model. Wang et al. [25] developed the amplification coefficient formula based on the idea of smeared crack, which was connected to the crack width. The difference method was employed to analyze the effects of the crack. Schultheiss et al. [26] further proposed the modified coefficient related to crack depth adaptation. These latest research achievements have promoted the understanding and analysis of the carbonation behavior of cracked concrete structures. Nevertheless, the carbonation-induced corrosion and its reverse influence on cracks were not considered.

For the RC beams under heavy loads, cracks provide a channel for accelerated carbonization, and the embedded reinforcements begin to rust prematurely due to de-passivation [27,28]. The corrosion effect will cause the development of bending cracks and the generation of longitudinal cracks in beams. The crack width continues to develop throughout the service life, further accelerating the carbonation of the concrete beam and the corrosion of the reinforcements. The coupling effect between beam cracking, concrete carbonation, and reinforcement corrosion synergistically accelerates structural degradation. Some new attempts have been made to address this issue, such as probabilistic [9] or machine learning approaches [29], as well as multi-scale or multi-physics modeling frameworks, which provide a valuable option for refined durability assessment. Fang et al. [30,31] conducted representative research work using the multi-phase field method to analyze the chloride ion diffusion field, the electrochemical corrosion of reinforcements, and the rust expansion cracking behavior. However, in their analysis model, the interaction among various physical fields is unidirectional, and the coupled effects of rust expansion cracks on chloride ion erosion have not been considered. Similar research was also conducted by Olawale et al. [32], Pan [33], Chen [34], and Qiu et al. [35], using different software. Luo et al. [36] introduced the coupling of crack propagation and chloride ionic transport to improve this model. This improvement reproduced the spalling phenomenon observed in physical experiments, while their model was a planar two-dimensional form, and the analysis object was a concrete specimen rather than a structure. Ozbolt et al. [37] proposed a finite element modelling method that considers cracking in transport processes for beam structures. However, their model still did not include the coupling effects of reinforcement corrosion on the cracks. Maekawa et al. [38,39] proposed a remarkable multi-physics coupling-based framework. They developed software to analyze the coupling problem between environmental effects and the cracks in a beam. However, most of their research focused on the erosion damage caused by chloride ions or sulfate ions, and the carbonation-induced corrosion deterioration was rarely seen. Overall, for a multi-physics field model, there were still three aspects that require further improvement. First, most research has focused on the influences of cracks on environmental erosion, but the reverse influences of corrosion on the development of cracks have not been considered. Second, most of the research subjects were concrete specimens rather than a concrete structure. Third, how to implement the multi-physics coupling-based framework with the general finite element programs?

To address these issues, a novel multi-physics coupling framework and implementation method are proposed in this paper. Compared to the previous study, three highlights can be found: (1) the explicit consideration of dynamic crack patterns and their feedback on carbonation–corrosion progression can be considered; (2) the model is of a three-dimensional style, which includes the integration of rust expansion effects on structural stiffness degradation; (3) the proposed method does not rely on computational software and can be conveniently implemented in general finite element software. With the proposed method, an 8 m RC beam in a heavy-haul railway is taken as an example, and the progression of carbonation–corrosion and stiffness degradation under different operational loads is analyzed. This study aims to elucidate the deterioration patterns and explore the influence mechanisms of environmental conditions and operational loads on the heavy-haul railway RC beams.

## 2. Multi-Physics Coupling-Based Analytical Method

### 2.1. Analysis Process

**Figure 1:**
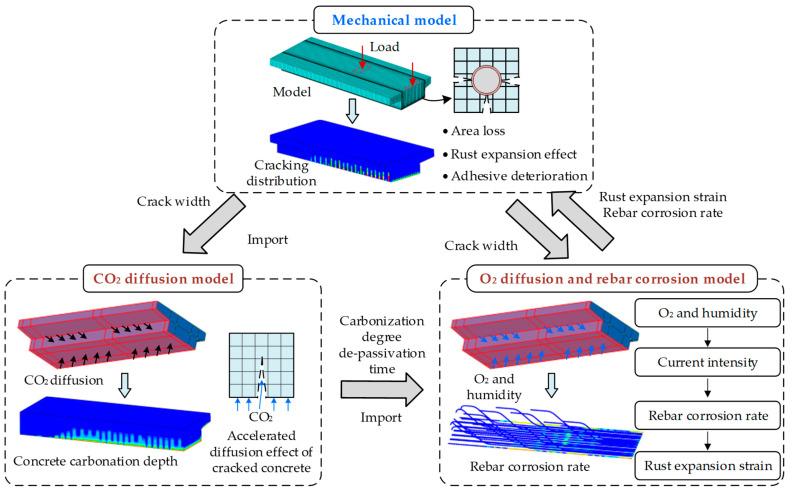
A framework for the proposed multi-physical field coupling analysis method.

The flowchart of the proposed multi-physics coupling analysis method for carbonation-induced corrosion deterioration of RC beams is shown in Figure 1. It mainly includes three types of models correspond to the simulation analysis of three physical fields: the mechanical analysis model is used to analyze the cracking state of the beam under service loads; the CO_2_ diffusion field analysis model is used to analyze the carbonation depth of the beam; the O_2_ diffusion and rebar corrosion model is used to analyze the corrosion rate of the steel bars as well as the corrosion-induced expansion effect.

The interaction mechanism and sequential coupling analysis process among the three types of analysis models are as follows:

(1) The service life of the bridge is divided into several time intervals. The sequential coupling analysis of each physical field is carried out at time intervals of *dt*, with *dt* varying from 1 to 5 years. The time interval can be adjusted according to the degree of change in the crack morphology. Within the *dt* intervals, the crack width was assumed to be constant and updated after the *dt* intervals. Even though small *dt* intervals will decrease the deviation, the calculation cost will increase significantly.
(2) Mechanical model analysis: The dead load and the operating train are taken as the applied loads to analyze the crack distribution and crack width of the beam. The crack width field variable is output and imported to the CO_2_ and O_2_ diffusion field for data interaction.
(3) CO_2_ diffusion model analysis: The carbonation depth of the beam within the *dt* interval is calculated based on the crack width of each element. The time when the steel bar surface reaches carbonation passivation is obtained and output as the de-passivation time field variable.
(4) O_2_ diffusion and rebar corrosion model analysis: The crack width field variable and the de-passivation time field variable of the steel bar surface are imported. The corrosion rate and corrosion-induced expansion strain of the steel bar within the *dt* interval are calculated based on the environmental temperature and humidity conditions, and then incorporated into the mechanical model.
(5) Repeat steps 2 to 4, and for each *dt* interval, the changes in beam cracking state, carbonation depth, and the steel bar corrosion rate are calculated in sequence. The variation laws of structural stiffness and cracking mode, with respect to time, are obtained to evaluate the durability of RC beams.

### 2.2. Mechanical Model

**Figure 2:**
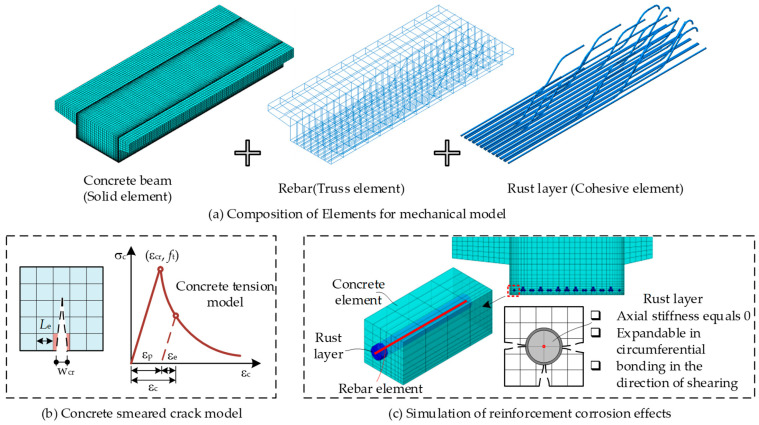
The finite element model for mechanical behavior analysis.

In the mechanical model, the concrete beam is modeled by solid elements, and the reinforcements are modeled using truss elements. Additionally, to simulate the bond degradation and rust expansion effect after rebar corrosion, the virtual rust expansion layers are set up based on the actual reinforcement bar dimensions and simulated by cohesive elements. The composition of the overall mechanical analysis model is shown in Figure 2.

(1) Concrete beam cracking analysis

A smeared crack non-linear constitutive model to account for tensile cracking and post-cracking effects of concrete solid elements. The uniaxial constitutive curve is depicted in Figure 2b. Before cracking, the concrete behaves linearly until the principal stress reaches its tensile strength. This stress threshold defines the onset of cracking. After cracking, the stress does not drop instantaneously to zero. Instead, it gradually decreases with strain due to the tension-stiffening effect between cracks. As suggested by Maekawa et al. [40], the softening phase follows a non-linear stress reduction characterized by an initially rapid decline followed by a slower rate, as formulated in Equation (1). Post-cracking, the crack width is estimated based on tensile strain using the empirical relationship provided in Equation (2). In the formulas, *E*_c_ represents the elastic modulus of concrete, *ε*_c_ is the strain of concrete, *ε*_cr_ is the peak tensile strain of concrete, *ε*_e_ is the elastic strain of concrete, *σ*_c_ is the stress of concrete, *f*_t_ is the tensile strength of concrete, *w*_cr_ is the width of concrete cracks, and *L*_e_ is the characteristic length of the concrete element in the cracking direction.

(1) $$\sigma_{c}=\left\{\begin{array}{cc} E_{c}\varepsilon_{c} & 0\leq \varepsilon_{c}\leq \varepsilon_{\mathrm{cr}}\\ f_{t}{\left(\varepsilon_{\mathrm{cr}}/\varepsilon_{c}\right)}^{0.4} & \varepsilon_{\mathrm{cr}}\leq \varepsilon_{c} \end{array}\right.$$

(2) $$w_{\mathrm{cr}}=\left(\varepsilon_{c}-\varepsilon_{e}\right)\cdot L_{e}$$

(2) Rebar corrosion effects

The effects of rebar corrosion include a reduction in effective cross-sectional area, corrosion-induced expansion strain, and degradation of the interface bond. They involve three different behaviors in different directions, which pose challenges for simulation. In this study, a novel modeling scheme is proposed, referred to as the “Truss + Virtual Cohesive” method, for a three-dimensional model to address this issue. The methodology is illustrated in Figure 2c, with the following implementation details:

(1) The nodes of rebar truss elements are rigidly connected to the adjacent nodes of the virtual rust layer element. The outer surface nodes of the virtual rust layer element are embedded into the concrete beam using an embedded constraint technique. Interactions between the rebar truss elements and the concrete beam are mediated through the virtual rust layer.
(2) The reduction in steel cross-sectional area is modeled by degrading the elastic modulus and yield strength of truss elements. A reduction factor *κ*_r_ is calculated using the corrosion rate *γ* via Xia and Jin’s formula [41], as shown in Equation (3). In the finite element software, a USDFLD field variable subroutine is programmed to achieve the purpose of material property degradation of the truss element.
(3) The UEXPAN subroutine is programmed to apply the radial expansion strain of the virtual rust layer element, whose material properties are orthotropic. The elastic modulus in the normal direction is assigned according to the rebar’s elastic modulus. The axial elastic modulus is set to zero to eliminate its effects on the beam’s flexural deformation.
(4) The bond deterioration caused by rebar corrosion is simulated by shear modulus reduction of the virtual rust layer, and the degradation factor *κ*_p_ is computed with corrosion rate *γ* using Xia and Jin’s formula [41], as shown in Equation (4).

(3) $$\kappa_{r}=1-\gamma$$

(4) $$\kappa_{p}=\left\{\begin{array}{cc} 1-11.875\gamma & \gamma <0.08\\ 0.05 & \gamma \geq 0.08 \end{array}\right.$$

### 2.3. CO_2_ Diffusion and Concrete Carbonization Analysis Model

(1) Concrete carbonation model

Based on the carbonation mechanism model proposed by Papadakis et al. [5] and research suggestions by Shi [23], the following assumptions are introduced to simplify the problem: (1) Concrete is a macroscopically homogeneous medium with stable and uniform local humidity; (2) The rate of change in CO_2_ concentration in the voids is much smaller than the carbonation reaction rate and can be ignored; (3) The hydration reaction is complete, and the rates of CH, CSH, C_3_S, and C_2_S formation in the later stage of hydration are ignored. Then the equilibrium equation for the concrete carbonation process can be established as shown in Equation (5):

(5) $$\frac{\partial }{\partial x}\left[D_{e}\frac{\left[{\mathrm{CO}}_{2}\right]}{\partial x}\right]=\frac{\partial }{\partial t}\left(\left[\mathrm{CH}\right]+3\left[\mathrm{CSH}\right]+3\left[C_{3}S\right]+2\left[C_{2}S\right]\right)$$

where M = [CH] + 3[CSH] + 3[C_3_S] + 2[C_2_S] represents the total amount of CO_2_ that can be consumed by the carbonate substances in the concrete. The mass diffusion analysis module is used to simulate the diffusion of CO_2_ and the carbonation process of concrete. The pH value of the pore solution in the concrete is calculated based on the CH concentration in the pore solution. A pH value of 7.5 is considered the complete carbonation limit, and a pH value of 12.5 is the uncarbonated limit. The pH range of 7.5 to 12.5 is used as the value range for the partially carbonated zone to assess the degree of carbonation of the concrete. When the pH value of the concrete at the surface of the reinforcement reaches the complete carbonation limit value, the storage time variable is taken as the time variable for the start of reinforcement corrosion.

(2) Diffusion coefficient of carbon dioxide in concrete

Papadakis et al. [42,43,44] proposed a calculation formula for the CO_2_ diffusion coefficient in concrete through mechanism analysis and experimental calibration, which has been widely applied in both academic and engineering fields. Li [11] further improved the formula by introducing correction coefficients for different humidity conditions, enhancing its applicability, as shown in Equation (6).

(6) $$\begin{array}{l} D_{e,{\mathrm{CO}}_{2}}=6.1\cdot 10^{-6}\epsilon_{p}^{3.0}\beta_{RH}\;\left(m^{2}/s\right)\\ \beta_{RH}=\left\{\begin{array}{cc} 5.46{\left(1-RH\right)}^{2.2}RH^{2.84} & RH\leq 55\%\\ {\left(1-RH\right)}^{2.2} & RH>55\% \end{array}\right.\\ \epsilon_{p}=\frac{\varepsilon }{\frac{c+kP}{\rho_{c}}+\frac{w}{\rho_{w}}}=\frac{\left(w-0.267\left(c+kP\right)\right)/1000}{\frac{c+kP}{\rho_{c}}+\frac{w}{\rho_{w}}} \end{array}$$

In Equation (6), *D*_e,co2_ represents the diffusion coefficient of CO_2_ in undamaged concrete; *ε*_p_ represents the porosity of concrete; RH is the relative humidity of the environment; *c*, *P*, and *w* respectively represent the cement, admixture, and water content in the concrete mix (kg/m^3^); *ρ*_c_ and *ρ*_w_ respectively represent the density of cement and water; and *k* is the admixture influence factor (0.3 to 1.0, 0.5 for low-calcium fly ash and 0.7 for high-calcium fly ash). The diffusion coefficient of undamaged concrete is calculated using Equation (6) based on the concrete mix parameters.

(3) Accelerated diffusion coefficient for cracked concrete

**Figure 3:**
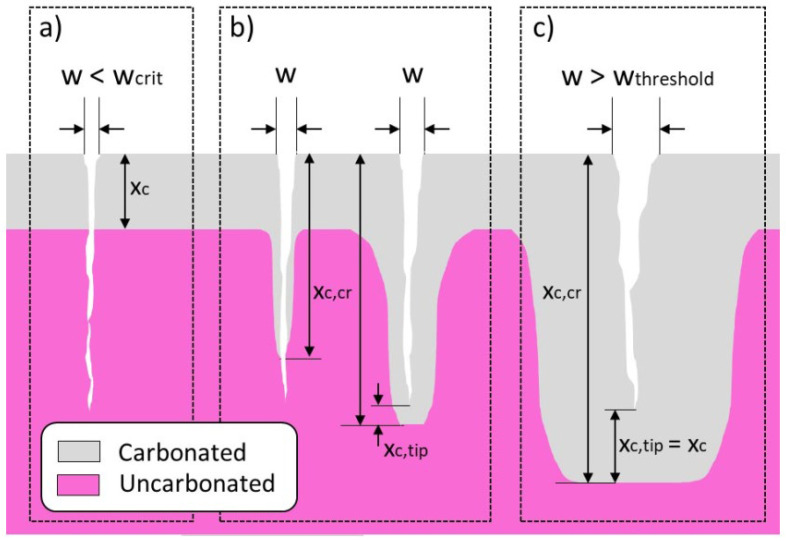
The accelerated carbonation phenomenon caused by concrete cracking [26]. (**a**) Crack width smaller than the critical crack width; (**b**) Crack width between critical crack width and threshold crack width; (**c**) Crack width exceed threshold crack width.

For the cracked concrete, the crack size is larger than the pore size, and the diffusion of CO_2_ in the crack area will accelerate the carbonation process [45], as shown in Figure 3. Wang [25] proposed an amplification factor of the diffusion coefficient of cracked concrete based on the concept of smeared cracks. The formula averaged the accelerated diffusion effect of cracked concrete within the element, which was suitable for implementation in a finite element model, as shown in Equation (7).

(7) $$\begin{array}{l} f\left(w_{cr}\right)=\frac{\left[D_{cr}\omega_{cr}+D_{0}(L_{e}-\omega_{cr})\right]}{D_{0}L_{e}}\approx 1+\frac{D_{cr}}{D_{0}}\frac{\omega_{cr}}{L_{e}}\\ \frac{D_{cr}}{D_{0}}=79,223-102,299\frac{w}{c} \end{array}$$

where *f*(*w*_cr_) is the accelerated diffusion coefficient of the cracked concrete, *D*_cr_ is the diffusion coefficient of the cracked concrete, and *D*_0_ is the diffusion coefficient of the uncracked concrete. In this paper, the accelerated carbonation effect at the crack is introduced according to Equation (7) for cracked concrete.

### 2.4. Rebar Corrosion Analysis Model

(1) The diffusion coefficient of oxygen in concrete

Papadakis et al. [42] derived the calculation formula for the diffusion coefficient of O_2_ in concrete based on the relationship between the molar mass and the diffusion coefficient of a gas. They proposed that the diffusion coefficient of O_2_ could be taken as 1.173 times that of CO_2_.

(2) Calculation of rebar corrosion ratio

The relationship between the volume loss of the rebar and the corrosion current density can be established as shown in Equation (8),

(8) $$\begin{array}{l} V_{\mathrm{effective}}=a\cdot \delta =\frac{A\cdot a\cdot i_{\mathrm{corr}}\cdot t}{z\cdot F\cdot \rho }\\ \delta =\frac{A\cdot i_{\mathrm{corr}}\cdot t}{z\cdot F\cdot \rho }\\ \gamma =\frac{4\pi V_{\mathrm{effective}}}{a^{2}}=\frac{4\pi \delta }{a}=\frac{2\delta }{R} \end{array}$$

where *t* (s) is the corrosion time; *F* = 96,500 (C/mol) is the Faraday constant; *z* is the valence of *Fe* in the reaction electrode; *A* = 56 g/mol and *ρ* = 7.86 × 10^−3^ g/mm^3^ are the atomic weight and density of *Fe* material, respectively; *a* (mm^2^) is the surface area of the steel reinforcement; *i*_corr_ (A/mm^2^) is the corrosion current density; *δ* and *γ* are the loss ratios of mean radius and volume of the corroded steel reinforcement, respectively.

The corrosion current intensity, *i*_corr,_ of steel reinforcement in an exposed normal atmospheric environment is calculated using the equation suggested by Ghods et al. [46], as shown in Equation (9). *r* is the resistivity of the concrete, and *C*_O2_ is the oxygen concentration. The resistivity *r* of concrete is calculated by the adopted formula of Li [11], as shown in Equation (10):

(9) $$i_{\mathrm{corr}\;}=-1.33\times 10^{-3}+\frac{3.00}{r}-3.83\times 10^{-4}\ln\left(C_{O_{2}}\right)+0.333\ln\left(C_{O_{2}}\right)/r$$

(10) $$r=\left[k\cdot \left(Cl^{-}-1.8\right)+100{\left(1-RH\right)}^{2}+40\right]\exp\left[3000\left(\frac{1}{T_{c}+273}-\frac{1}{298}\right)\right]$$

where *k* is the coefficient related to the water–cement ratio of concrete (*k* = −11.1 when *w/c* = 0.3~0.4; *k* = −5.6 when *w/c* = 0.5~0.6), *Cl^−^* is the chloride ion content (percentage of cement weight), and RH is the environmental relative humidity (%). *T*_c_ is the ambient temperature in Celsius (°C).

(3) Rust expansion strain of rebar

With the specified rebar volume loss rate *γ*, the free expansion strain of the corrosion products can be calculated according to the engineering strain definition as Equation (11). The average stiffness of the corrosion products and the original reinforcing bars can be calculated using Equation (12):

(11) $$\varepsilon_{\mathrm{free}}=\sqrt{1+\gamma \left(\alpha -1\right)}-1$$

(12) $$E_{s,\mathrm{eq}\;}=\frac{1+\gamma (\alpha -1)}{\left(1-\gamma /E_{s}\right)+(\gamma \alpha /G)}$$

where *E*_s_ is the elastic modulus of the original reinforcing bars (200 GPa), and *G* is the elastic modulus of the corrosion products (7 GPa).

### 2.5. Model Validation

**Figure 4:**
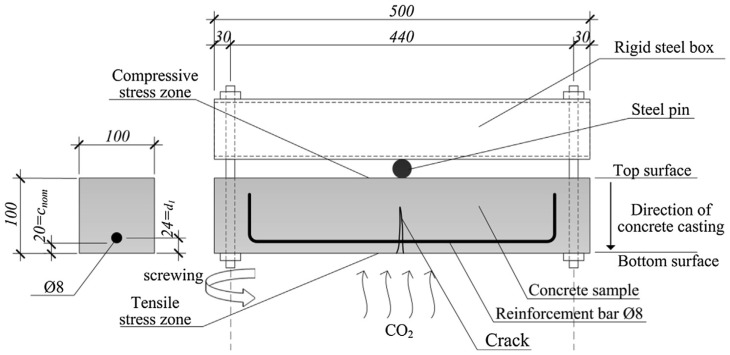
Experiment setup of carbonation-induced corrosion of RC beam.

Carević and Ignjatović [47] conducted a complete test on the cracking, carbonation, and corrosion processes of reinforced concrete specimens. In their tests, the prismatic RC samples were first loaded until the specified crack width (0.05, 0.10, 0.20, or 0.30 mm) was reached, and then subjected to accelerated carbonation and corrosion tests lasting 28 days. The effects of cracks on the carbonation depth were compared, and their influence on the corrosion of cracked and uncracked samples was analyzed by observing the reduction in the reinforcement cross-section. Figure 4 shows the experiment setup by Carević and Ignjatović [47], which is employed to discuss the applicability of the proposed method.

The dimensions of the reinforced concrete prisms were 100 × 100 × 500 mm, with an 8 mm diameter reinforcing bar embedded. The 28-day mean cube compressive strength and elasticity modulus of concrete were 34.7 MPa and 31.9 GPa, respectively. The accelerated carbonation and corrosion tests were conducted over 28 days at a CO_2_ concentration of 2%, a relative humidity (RH) of 65 ± 5%, and a temperature of 20 ± 2 °C in a carbonation chamber.

**Figure 5:**
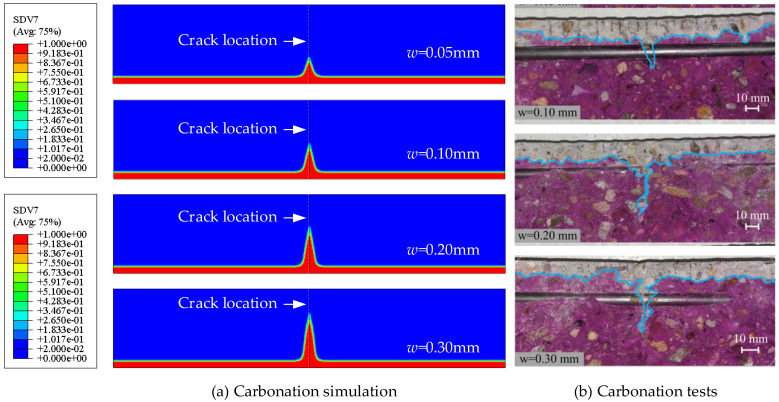
Comparisons of carbonation of cracked concrete between simulation and tests.

**Figure 6:**
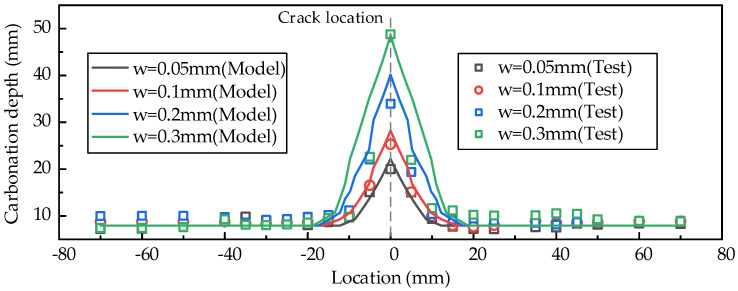
Carbonation depth distributions around the crack locations.

**Figure 7:**
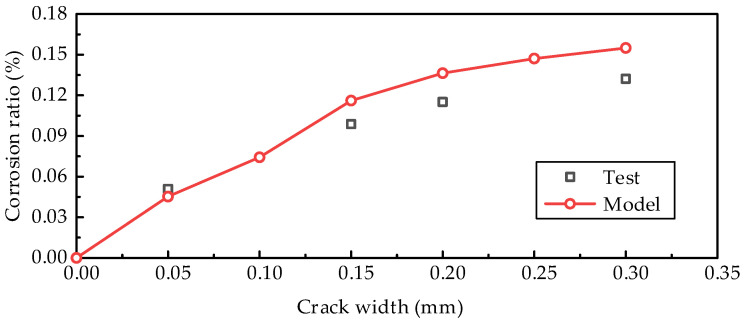
Volume corrosion ratio of reinforcements with different crack widths.

The loading cracking, concrete carbonation, and steel bar corrosion processes are simulated with the proposed method. Figure 5 shows the comparisons of concrete carbonation within the specimens with different crack widths. The carbonation depth at the crack section is significantly greater than in the uncracked zones. Figure 6 illustrates the carbonation depth distributions around the crack locations, as well as the comparisons between the simulation results and the test data. For the uncracked zones, the carbonation depth is approximately 8 mm; however, it increases sharply as the crack is approached. The simulated results for the maximum carbonation depth are generally consistent with the test results, with a maximum deviation of 18.36% in overestimation. The maximum overestimation deviation between the simulated and measured values of the steel bar corrosion rate is 17.22% (the case with 0.10 mm crack width is excluded due to its apparent deviation from the other cases), as shown in Figure 7. The variability of concrete carbonation and corrosion may cause the deviations. In the numerical model, the equations by Papadakis et al. [42,43,44] are adopted to calculate the carbonation depth. The deviations inevitably exist due to the influence of uncertain factors, such as local deviations in aggregate distribution and mix proportion. The calculated carbonation depth is slightly greater than the test results, resulting in a longer corrosion time and a higher corrosion ratio of the reinforcements.

Nevertheless, the variation pattern of the steel bar corrosion rate with the crack width exhibits a high degree of consistency between simulation and experimental results. The corrosion rate of reinforcing bars shows a trend of increasing with the increase in crack width, but the growth slope gradually decreases. The mechanism of this phenomenon can be explained as follows: a larger crack width leads to earlier corrosion of the steel bars, and the increase in corrosion time results in a higher corrosion rate; however, when the crack width increases to a certain value, the crack depth exceeds the effective height of the steel bars, and the sensitivity of the steel bars’ corrosion initiation time to the crack width decreases. The comparisons in Figure 6 and Figure 7 demonstrate that the proposed method effectively captures the impact of concrete cracking on carbonation and steel bar corrosion, which are crucial in the multi-physics field coupling analysis.

## 3. Durability Assessment of Reinforced Concrete Beam in Heavy-Haul Railways

### 3.1. Structural Details

(1) Structural design parameters

**Figure 8:**
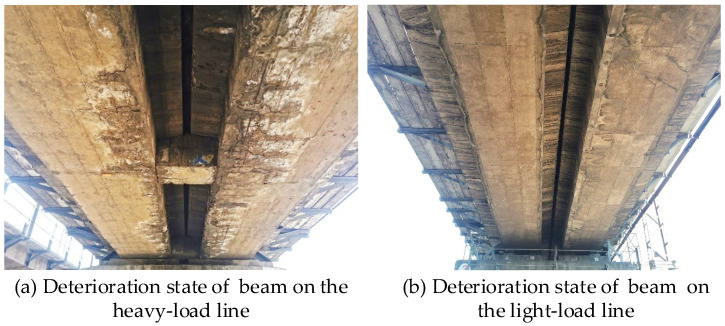
Deterioration state of the concrete beam in heavy-haul railways.

The 8 m-span low-height RC beam is a representative beam type in China’s heavy-haul railway bridges. To date, it has been in operation for nearly 40 years. Based on the on-site exploration, the typical carbonation and corrosion deterioration diseases have emerged, as shown in Figure 8. Taking the 8 m-span low-height RC beam as an example, the deterioration patterns of the girder under different operational train loads are analyzed.

**Figure 9:**
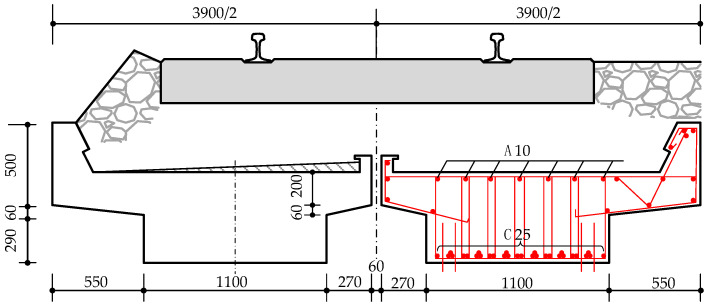
Dimension details of the cross-section of the RC beam.

The typical cross-section of the beam is shown in Figure 9. The cross-section is 550 mm in height and 1100 mm in width at the bottom. The outer cantilever length is 550 mm, and the inner cantilever length is 270 mm. There are 31 rebars arranged at the bottom of the beam, with a protective layer thickness of 31 mm. These rebars are made of 16Mn with a diameter of 25 mm. The beam was built according to the old design code. According to the design and construction documents, the cube’s compressive strength is 35 MPa, and the concrete mix ratio is 1:1.61:2.98:0.46 (cement/sand/stone/water).

(2) Operating loads

**Figure 10:**
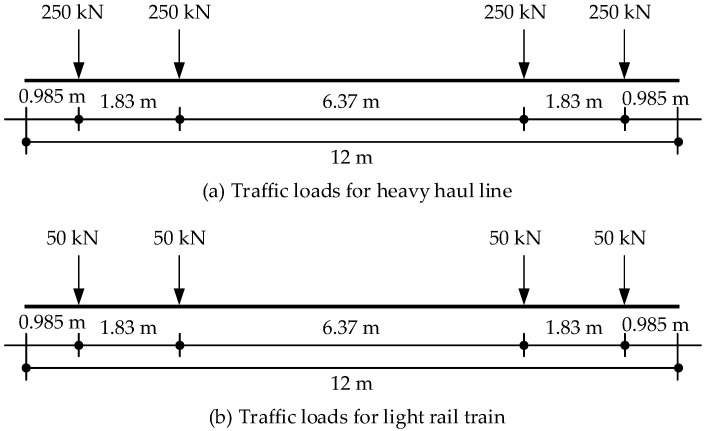
Load diagram for heavy haul line and light rail train.

The self-weight of the beam is 2.11 t/m, and the dead load of the ballast and the sidewalk is calculated as 1.96 t/m. The load diagrams of the heavy-haul line and light rail train are shown in Figure 10a,b, respectively. The live load of the train is applied at the most unfavorable bending moment at the mid-span, and the impact coefficient is taken as 1.316.

(3) Service environmental parameters

For the normal atmospheric conditions at the bridge location, the volume fractions of CO_2_ and O_2_ in the atmosphere are 0.04% and 21%, respectively. The ambient temperature is considered to be 25 °C, and the ambient humidity at the most unfavorable carbonation condition is RH = 55%. Although the temperature and humidity in the actual environment change daily, this would make the simulation extremely complex. Therefore, in this study, the average temperature and the most unfavorable humidity conditions are adopted.

(4) Carbonation depth calculation under uncracked conditions

**Figure 11:**
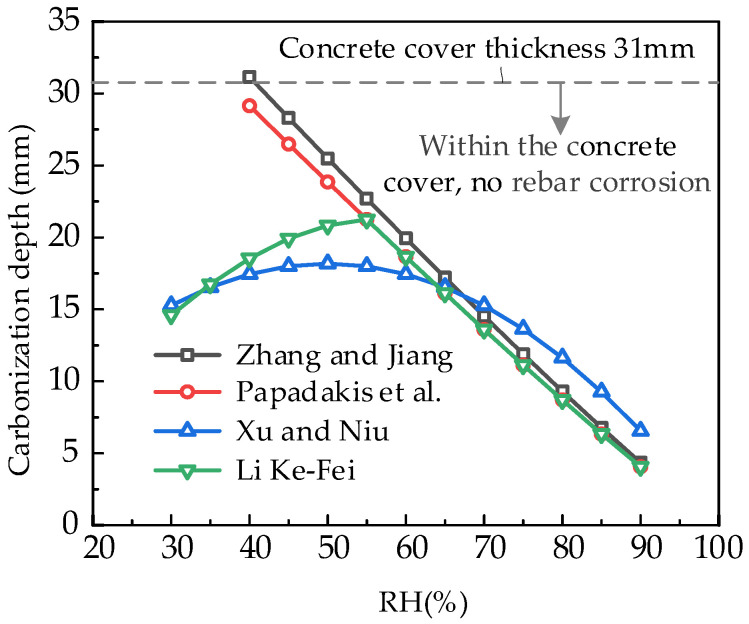
Carbonation depth calculation without considering cracking [11,43,48,49].

Based on the concrete mix ratio, the carbonation depth of concrete within a 100-year service life is first calculated using the standard equation, assuming no cracking. The formulas by Zhang and Jiang [48], Papadakis et al. [5], Xu and Niu [49], and Li [11]. The results are shown in Figure 11. The results indicate that:

(1) The calculation results of Zhang and Jiang [48] and Papadakis et al. [5] are generally close. For low-humidity conditions, the results from these two equations are relatively large. Li [11] modified the equation of Papadakis et al. [5] for the low-humidity conditions, and the calculated carbonation depth reaches its maximum (approximately 22 mm) at a relative humidity of 55%. The calculated carbonation depth, as determined by Xu and Niu’s formula [49], is less than 18 mm, with humidity conditions ranging from 30% to 90%.
(2) When the relative humidity range is 40% to 80%, the calculated carbonation depth results of uncracked concrete are all smaller than the thickness of the protective layer, 31 mm within 100 years. The results indicate that the reinforcement within the beam should not experience de-passivation and corrosion if the cracking effects are ignored. This result is inconsistent with the actual corrosion and deterioration of bridges, as shown in Figure 8. The multi-field coupling analysis method should be adopted to account for the cracking of the beam in a refined analysis of carbonation and corrosion deterioration.

### 3.2. Durability Assessment by Multi-Physics Coupling-Based Analytical Method

Based on the proposed multi-physics coupling-based analytical method, a sequential coupling analysis is conducted to examine the interactions between beam cracking, concrete carbonation, and reinforcement corrosion. The discrete time step was set at 5 years, and the deterioration of structural stiffness was analyzed and evaluated over a 100-year service life.

#### 3.2.1. Comparisons of the Initial Cracking of the Beam

**Figure 12:**
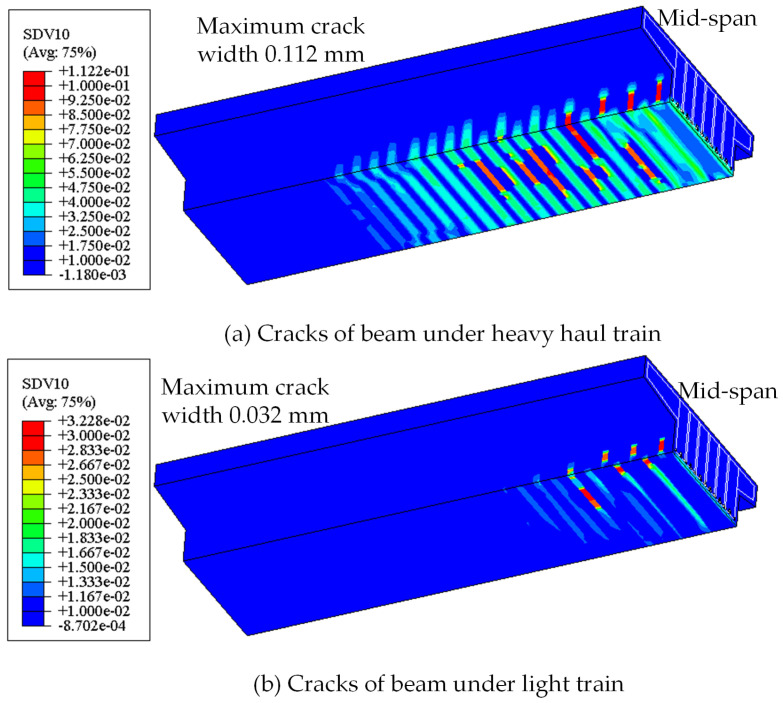
Cracking comparisons under different loading conditions.

With the mechanical model described in Section 2.2, the initial crack patterns of the beam under the first loading of heavy and light train loads are analyzed, respectively. The results are shown in Figure 12. The crack distribution range of the beam in the heavy line is wider, with the maximum crack width reaching 0.112 mm, while the beam in the light rail line only cracks in a local range at the mid-span, with the maximum crack width being only 0.032 mm.

#### 3.2.2. Comparison of Concrete Beam Carbonation

**Figure 13:**
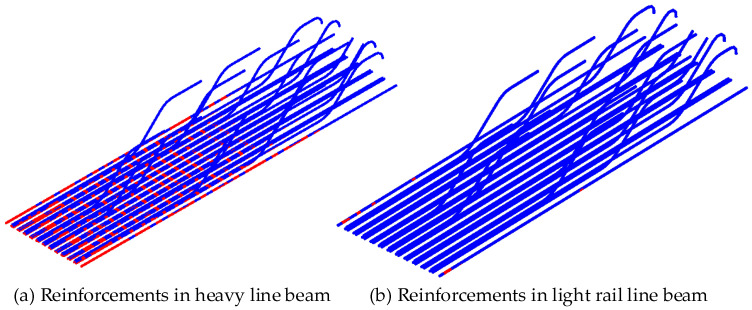
Comparison of concrete carbonation on the rebar surface.

Figure 13 shows the comparison of concrete carbonation on the surface of rebars after 100 years of deterioration. The red areas in the figure indicate that the concrete on the rebar surface has carbonated and reached a state of corrosion. The beam in a heavily trafficked line has a wide cracking range and a large crack width; hence, the carbonation depth reaches the rebar surface approximately 8 years after the cracking occurs. For the beam in a lightly trafficked line, the carbonation depth reaches the rebar surface, resulting in cracking approximately 20 years after the construction. The location of the de-passivated rebars is consistent with the initial crack location. After the passivation, the rebars start to rust locally. However, the rebars at other places have not reached the carbonation passivation condition.

#### 3.2.3. Comparisons of Crack Patterns with Time Variation

**Figure 14:**
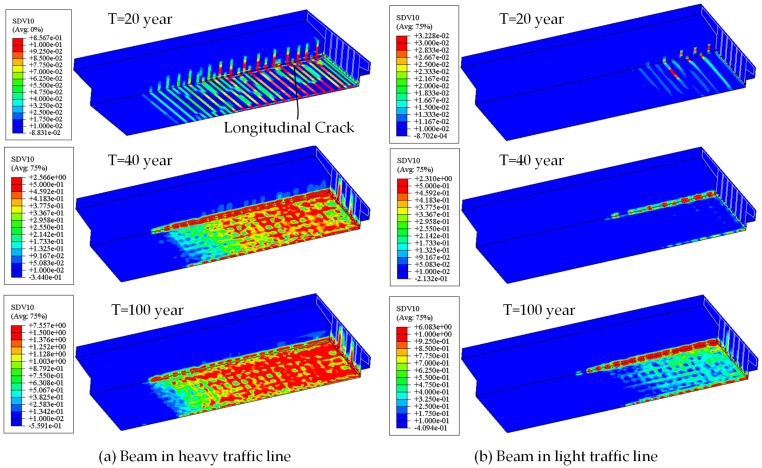
Comparisons of crack patterns with time variation.

The comparison of crack patterns in the beam between the light and heavy traffic lines is shown in Figure 14.

Under heavy traffic loads, vertical cracks are formed first, and then the corner reinforcements passing through the cracks begin to rust. The rust expansion effect causes the appearance of “short” longitudinal cracks at the vertical crack locations. Meanwhile, the width and height of the vertical cracks also develop. With the increase in the corrosion ratio and duration, the “short” longitudinal cracks gradually extend along the longitudinal direction. Then, the longitudinal and vertical cracks peel off the protective layer from the concrete beam, weakening the connection and reducing the structural stiffness.

For beams in the light traffic line, longitudinal rust expansion cracks only occur at the corner positions. The cracks in the other zones are not obvious. The simulated crack patterns are similar to those observed in practice, as shown in Figure 8.

#### 3.2.4. Comparison of Structural Stiffness Degradation

**Figure 15:**
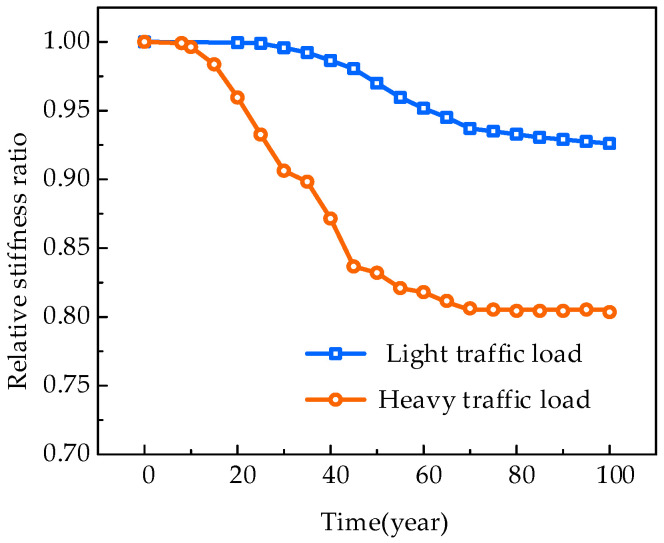
The deterioration curves of the relative stiffness ratio of the beam.

For each time step, the train load is applied at the most unfavorable position of the mid-span bending moment. The deflection caused by the live load is taken to evaluate the structural stiffness. The deflection ratio of the uncorroded beam to that of the corroded beam is defined as the relative stiffness ratio and used to assess the structural stiffness deterioration. Figure 15 illustrates the deterioration curves of the relative stiffness ratio for the beam under different loading conditions.

The stiffness of the beams on both the light and heavy train lines decreased over time. For the beam in the heavy train line, the structural stiffness decreases by approximately 0.4%, 4.0%, 12.9%, and 20.0% for service times of 10, 20, 40, and 100 years, respectively. For the beam in the light train line, corrosion occurs only at the corner reinforcements across the cracks. The degree of reduction in structural stiffness is relatively small, with decreases of 0.0%, 0.1%, 1.4%, and 7.4% in structural stiffness within 10, 20, 40, and 100 years, respectively.

## 4. Discussion

(1) Influence of corrosion effects on the structural stiffness

**Figure 16:**
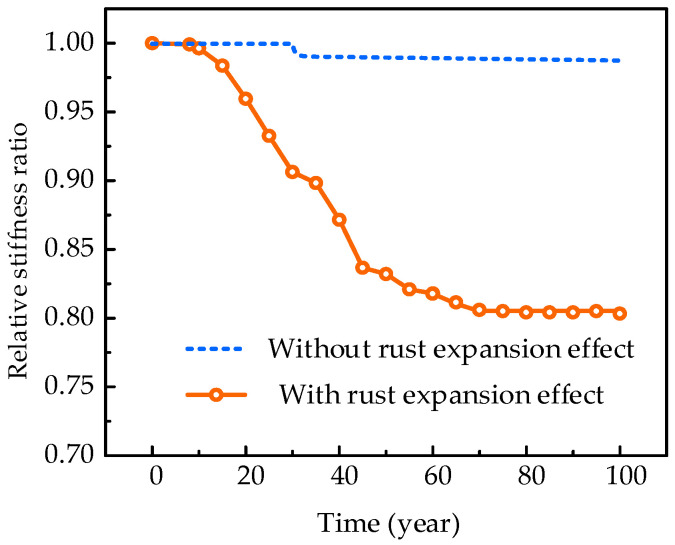
Influence analysis of the rust expansion effect on the structural stiffness.

A new analysis condition is set to beam in the heavy-haul line to discuss the influences of three types of corrosion effects—namely, the reduction in steel area, the deterioration of interface bonding, and the rust expansion effect—on structural stiffness deterioration. During the analysis procedure, the impact of rust expansion is ignored, and the calculated results are presented in Figure 16. The results indicate that for the analysis of structural stiffness in the early cracking stage, the rust expansion effect is the leading cause of structural performance deterioration. Without considering the impact of rust expansion, the change in structural stiffness is negligible. Assuming a steel corrosion rate of 1%, the effective area of the steel remains 99% of its original area, and its stiffness change is negligible. However, at this time, the free expansion strain caused by steel corrosion can be estimated at 0.009, which will generate a relatively large expansion stress and cause concrete cracking.

The mechanism of the rust expansion effect on the deterioration of structural stiffness can be analyzed as follows: For cracked reinforced concrete beams without corrosion, the protective layer of concrete in the tension zone still contributes to the beam’s stiffness due to the tensile stiffening effect. As the rust expansion effect intensifies, the rust expansion cracks weaken the interaction between the protective layer and the upper concrete. In severely rusted areas, it can be considered that the protective layer has peeled off, and the contribution of the protective layer to the structural stiffness gradually decreases to zero, resulting in a reduction in structural stiffness. As the steel corrosion rate continues to increase, the steel will experience a significant reduction in its effective area and a decrease in yield strength, which will impact its structural integrity and safety.

(2) Influence of the key parameters

The factors influencing the durability of concrete structures are complex and multifaceted. With the proposed model, the simulation results are sensitive to the key parameters (e.g., crack width, concrete cover, and environmental conditions). In this study, a simplified scenario with constant temperature and humidity is employed to explore the applicability of the multi-physics coupling-based evaluation method. The influence of environmental temperature and humidity on stiffness degradation is discussed in this section.

**Figure 17:**
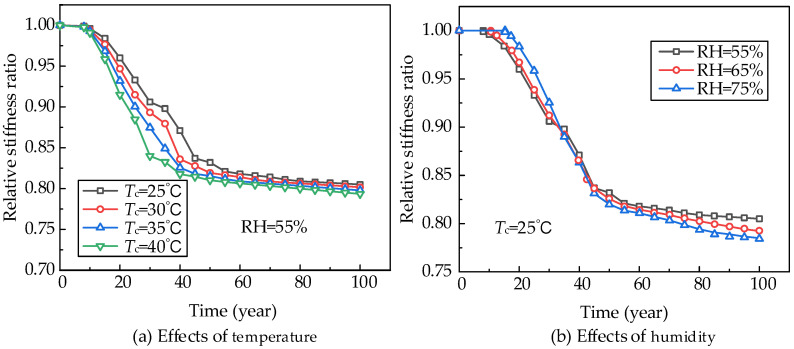
Influence analysis of environmental temperature and humidity.

The effects of environmental temperature on concrete carbonation are usually considered limited [50]. However, the temperature will increase the chemical corrosion reaction rate of steel bars, thereby accelerating their rusting. In the proposed model, the temperature effect is considered by the resistivity of concrete, as shown in Equation (10). By changing the temperature from 25 to 40 °C, the comparisons of the relative stiffness ratio between different temperature cases are shown in Figure 17a. It can be observed that the stiffness degradation ratio increases with rising temperature. However, if the temperature [51] were larger than 40 °C, this pattern would change, as high temperatures would also slow down the diffusion of O_2_, thereby decreasing the corrosion ratio.

The effects of ambient humidity on the carbonation-induced corrosion can be divided into two aspects. First, the larger ambient humidity increases the pore saturation in concrete, thereby decreasing the diffusion ratio of CO_2_ and the carbonation process. Second, once the reinforcing bars reach the stage of de-passivation and start to rust, the larger ambient humidity will increase the chemical corrosion reaction rate. Changing the ambient humidity RH from 55% to 75%, four cases are analyzed and compared in Figure 17b. It can be seen that the case with RH = 75% begins to rust at 15 years, later than the case with RH = 55%. However, once the conditions for steel bar corrosion are met, the case of RH = 75% shows a higher corrosion and stiffness degradation ratio.

(3) Discussion on the proposed model

The proposed method offers a clear advancement by integrating the crack evolution, carbonation, and corrosion processes in a sequentially coupled framework. The novelty lies in the following: (1) the explicit consideration of dynamic crack patterns and their feedback on carbonation–corrosion progression; (2) the integration of rust expansion effects on structural stiffness degradation; (3) the proposed method that does not rely on computational software and can be conveniently implemented in general finite element software.

However, due to the complexity of concrete structure durability, some limitations require further improvement. (1) In this model, the environmental conditions are simplified to be constant according to the average temperature and humidity. The seasonal or microclimatic variations in real railway environments have not been considered in current research, such as the conditions of alternating dry and wet cycles. (2) The sequential coupling approach is adopted in this paper to simplify the simulation. However, simultaneous interactions in certain conditions, where the crack growth, carbonation, and corrosion could occur concurrently at similar timescales, are neglected. (3) The field data validation of the proposed model is still needed in further study.

In future work, improvements are still needed in the following aspects. (1) More complex environmental conditions and loading cases, such as the changing temperature and humidity conditions, the erosion effect of chloride ions and sulfate ions, as well as their interactions with fatigue-cracking, etc., can be included. (2) The sequentially coupled framework can be further improved by the co-simulation technology of multi-field to consider the transient coupling effect of cracks and environmental parameters. (3) The durability comparison analysis for the beams with different strengthening techniques.

Regardless, the simulation results obtained using the proposed model accurately reflect the degradation mechanism of structural stiffness under the coupling conditions of heavy load and environmental action. The findings from the simulations can provide guidelines for the durability maintenance of beams in heavy-haul railways. The simulation reveals that the structural crack width plays a significant role in the deterioration of structural stiffness, owing to its accelerated diffusion effect. Inspecting the crack width is crucial for maintaining heavy-haul railway infrastructure. For beams with significant cracks, simple repairs or surface coatings are insufficient to control crack propagation. The cross-section enlargement method using concrete or steel plates may be more effective. For concrete beams in newly constructed structures, it is recommended to use prestressed concrete beams as much as possible to prevent cracks from occurring.

## 5. Conclusions

A multi-physics field coupling-based analysis method is proposed in this paper for evaluating the carbonation and corrosion-induced deterioration of reinforced concrete beams in heavy-haul railways. The interactions among beam cracking, concrete carbonation, and rebar corrosion are considered. A set of accelerated carbonation and corrosion tests was conducted on cracked RC beams to verify the effectiveness of the method. A full-scale 8 m RC beam is studied to analyze the time-dependent development of cracks, concrete carbonation, and rebar corrosion. The effect of operational train loads on the beam’s stiffness degradation was also examined. The main conclusions are as follows:

(1) The proposed method effectively captures the accelerated carbonation at beam cracks. The predicted carbonation depth distribution and the relationship between reinforcement corrosion rate and crack width align well with the results of the accelerated indoor tests. The maximum overestimation deviations are 18.36% and 17.22%, respectively.
(2) Within a 100-year service life, the maximum relative stiffness reduction of the 8 m RC beam is approximately 20.0% in the heavy traffic line and 7.4% in the light traffic line. The stiffness degradation is closely linked to the cracks caused by the operational load. Reinforcement rust expansion-induced cracking of the concrete beam’s protective layer reduces its contribution to structural stiffness, which is the leading cause of stiffness degradation in reinforced concrete beams. As the protective layer weakens, the structural stiffness degradation rate generally follows a “first increase then decrease” trend.
(3) Inspecting the crack width is crucial for maintaining heavy-haul railway infrastructure. For beams with significant cracks, simple repairs or surface coatings are insufficient to control crack propagation. The cross-section enlargement method using concrete or steel plates may be more effective.
(4) The proposed method offers an advanced option by integrating the crack evolution, carbonation, and corrosion processes in a sequentially coupled framework. This framework is employed in this paper to simulate the carbonation and corrosion process under constant temperature and humidity conditions. Further research is still needed in the future, including studies on variable temperature and humidity conditions, chloride-induced corrosion, and fatigue-cracking interactions.

## Data Availability

The original contributions presented in this study are included in the article. Further inquiries can be directed to the corresponding author.
